# Impacted Fracture of the Femoral Neck

**Published:** 1880-03-20

**Authors:** A. F. Kinne

**Affiliations:** Michigan


### *A CASE OF IMPACTED FRACTURE OF THE FEMORAL NECK.*

BY A. F. KINNE, A.M., M.D., OF MICHIGAN.

The following case will serve at least to illustrate the point, that a fractured femoral neck may be so firmly impacted as to bear all the handling necessary to the making of a thorough diagnosis, and a good deal of rough shaking up besides, without being broken up or weakened.

Mrs. H. C., aged about 46, healthy, and in very good flesh without being corpulent, was alighting from a high "coal-box" buggy, at the door of a church, four miles from home, when, in some way, her feet become entangled in the skirts of her dress, and she was precipitated, head foremost, to the ground.

A little son, upon whom she was depending for assistance, was too small a boy for such a service; he managed, however, as well as he could, to keep her head from injury, and thus it came about that her hips came to the ground first and received the main shock of the fall. And when the bystanders who sprang to her aid had raised her to her feet again, it was found that the right hip was wholly disabled. She could not walk and she could bear no weight upon that foot.

Feeling sure, therefore, that her right hip had suffered a serious injury of some kind, she was lifted, under her own direction, up into the buggy again, and, in a sitting posture, the only one of which the vehicle admitted, conveyed back again to her own house. And there a messenger, driving more than twice as fast as she did, enabled me to meet her and get ready a fracture bed before she was taken from the buggy.

But the matter of immediate interest in such a case as this is not the diagnosis itself, but the *making* of it. For an impaction is a great advantage in the case of a broken hip. And if this condition should be found existing—and the circumstance of the accident pointed strongly towards the possibility of it—the greatest care should be taken to handle the limb, if possible, in such a way as not to disturb it.

Upon uncovering the feet I found the right leg short, say about half an inch, and the right foot everted, not much, but it certainly was turned outwards unnaturally. And the concurrence of these two deformities settled the point that the lesion in question was a fracture and not a dislocation. And just here was the point where the question must be settled, and by the sense of touch alone, whether the fracture was an impacted one or not. Were these deformities *permanent*, or could the eversion and the shortening be easily made a little more or a little less?

By a careful comparison of the two limbs I satisfied myself that they were alike. One of the feet could be everted a little more than the other, and inverted a little less; but the sense of resistance was the same; they *felt* alike, and the fracture was therefore an impacted one.

But there is sometimes a way of testing a question that is already settled. Upon uncovering the hips I found the right one flattened, about the thickness of the hand, and upon rotating the limb it did not turn upon the axis of the femur; the trochanter flopped.

In the treatment of this case, I used a modified Desault's splint, making extension by means of Dixicrosby's long strips of adhesive plaster, and counter extension with a perineal band—the indications being to support the limb and prevent the impaction from being disturbed by the action of their femoral muscles.

My objection to the action of a pulley and weight is that it never sleeps, while the muscles are perfectly quiescent the greater part of the time. It is, therefore, unnecessarily fatiguing and calculated to produce abrasions and consequent discomfort. And I think, moreover, that it is more likely to weaken and break up an impaction than the plan which I adopted. And especially is all this true if the surface of the bed is made to incline towards the head, so as to prevent the patient's body from gravitating towards the points of fracture and of peri neal pressure.

I was greatly assisted in this case by the intelligent cooperation of the patient herself and of her husband. And in six weeks the disabled hip was strong enough to do without support, and my patient was permitted gradually to get upon her feet again and recover the use of her limbs.

About a year and a quarter have now elapsed since this accident happened, and the eversion and the shortening remain, apparently, just as they were when this case was taken in hand; the difference in the two foot heels is not more than half an inch, and but for some stiffness of the joint—not yet overcome—the patient thinks she could walk without a limp.

